# Women’s empowerment and female genital mutilation intention for daughters in Sierra Leone: a multilevel analysis

**DOI:** 10.1186/s12905-021-01340-2

**Published:** 2021-05-13

**Authors:** Edward Kwabena Ameyaw, Seun Anjorin, Bright Opoku Ahinkorah, Abdul-Aziz Seidu, Olalekan A. Uthman, Mpho Keetile, Sanni Yaya

**Affiliations:** 1 School of Public Health, Faculty of Health, University of Technology Sydney, Ultimo, NSW Australia; 2 Warwick Centre for Applied Health Research and Delivery (WCAHRD), Division of Health Sciences, Warwick Medical School, University of Warwick, Coventry, CV4 7AL UK; 3 Department of Population and Health, College of Humanities and Legal Studies, University of Cape Coast, Cape Coast, Ghana; 4 College of Public Health, Medical and Veterinary Services, James Cook University, Douglas, Australia; 5 Population Studies and Demography, University of Botswana, Gaborone, Botswana; 6 School of International Development and Global Studies, Faculty of Social Sciences, University of Ottawa, 120 University Private, Ottawa, ON K1N 6N5 Canada; 7 The George Institute for Global Health, Imperial College London, London, UK

**Keywords:** Female Genital Mutilation/Cutting, Women’s intentions, Cutting daughters, Multi-level analysis, Sierra Leone

## Abstract

**Background:**

Female genital mutilation is common in Sierra Leone. Evidence indicates that empowering women provides protective benefits against female genital mutilation/cutting (FGM/C). Yet, the relationship between women’s empowerment and their intention to cut their daughters has not been explored in Sierra Leone. The aim of this study was to assess the association between women’s empowerment and their intention to have their daughters undergo FGM/C in the country.

**Methods:**

Data for this study are from the 2013 Sierra Leone Demographic and Health Survey. A total of 7,706 women between the ages of 15 and 49 were included in the analysis. Analysis entailed generation of descriptive statistics (frequencies and percentages), and estimation of multi-level logistic regression models to examine the association between women’s empowerment, contextual factors and their intentions to cut their daughters.

**Results:**

A significantly higher proportion of women who participated in labour force reported that they intended to cut their daughters compared to those who did not (91.2%, CI = 90.4–91.9 and 86.0%, CI = 84.1–87.8, respectively). Similarly, the proportion intending to cut their daughters was significantly higher among women who accepted wife beating than among those who rejected the practice (94.9%, CI = 93.8–95.8 and 86.4% CI = 84.9–87.8, respectively). A significantly higher proportion of women with low decision-making power intended to cut their daughters compared to those with high decision-making power (91.0%, CI = 89.0–92.8 and 85.0% CI = 82.2–87.4, respectively). Results from multivariate regression analysis showed that the odds of intending to cut daughters were significantly higher among women who participated in labour force (aOR = 2.5, CI = 1.3–4.7) and those who accepted wife beating than among those who did not (aOR = 2.7, CI = 1.7–4.5). In contrast, the likelihood of intending to cut daughters was significantly lower among women with high than low knowledge (aOR = 0.4, CI = 0.3–0.7), and among those aged 45–49  than among those aged 15–19  (aOR = 0.2, CI = 0.0–0.6).

**Conclusion:**

The findings underscore the need to align anti-FGM/C policies and programmes to women who have undergone FGM/C, those with low knowledge, women who support wife beating and young women. Such interventions could highlight the adverse implications of the practice by stressing the psychological, health and social implications of FGM/C on its survivors.

## Background

The third and fifth Sustainable Development Goals (SDGs) aim to achieve good health as well as attain gender equality [1]. Attaining these goals is likely to remain a challenge in countries that practice female genital mutilation/cutting (FGM/C), which is a gross violation of the rights of women and girls [2]. According to the World Health Organization (WHO), FGM/C involves all procedures such as partial or total elimination of the external genitalia or any injury to the female genital organ for non-medical reasons [2]. Several global, regional and national level policies and legislative instruments address FGM/C. For instance, the WHO in collaboration with the United Nations Children’s Fund (UNICEF) and the United Nations Population Fund (UNFPA) issued a joint statement against the practice in 2007 [3]. The UN General Assembly also passed a resolution on the elimination of FGM/C in 2012 [4].

Over 26 countries in Africa have instituted measures to end the practice [5]. Twenty-four of these countries have instituted legislative instruments to end the practice. For instance, countries such as Djibouti (1995), Ghana (1994), Burkina Faso (1996), Ivory Coast (1998), Senegal (1999), and Togo (1998) have banned FGM/C [6]. Nonetheless, such efforts may have limited impact in societies and countries where about 90% of women in reproductive ages have undergone the practice [7]. However, evidence from the Demographic and Health Surveys (DHS) shows a trend towards change, with the practice being higher among older than younger women [7].

Women’s intention to subject their daughters to FGM/C has greater implications for the success or otherwise of the global community’s efforts to end the practice. While empowered women may be resilient in advocating for and protecting their wellbeing and that of their children, unempowered women may ensure the perpetration of FGM/C [8]. Women’s empowerment, being multi-dimensional, has been measured by various indicators. Indicators such as decision-making capacity, justification of sexual violence, labour force participation and one’s level of knowledge and education are some of the commonly used indicators for conceptualising women’s empowerment [8–11]. Women’s empowerment has been widely acknowledged to enhance women's decision-making capacity and boost their negotiation skills [12, 13].

FGM/C is endemic in Sierra Leone due to cultural interpretations, societal acceptance and underlying religious drivers [14, 15]. Whereas feminists and some scholars argue that FGM/C serves as a tool to subordinate and control women [16, 17], in the case of Sierra Leone, women themselves organise and ensure that FGM/C is carried out unabated [15, 18]. In Sierra Leone, belonging to the FGM/C practicing group called the ‘Bondo Society’ and undergoing the practice earn women accolades such as being well brought up, marriageable as well as having an enhanced public recognition [18, 19]. The prowess and complexities of the “Bondo Society” is further strengthened by the patriarchal nature of the practicing communities in Sierra Leone [20].

Women’s role in perpetuating the practice of FGM/C in Sierra Leone raises questions about the extent to which their empowerment may be associated with their intentions to continue or abandon the practice in light of the evidence that empowered women are less likely to support continuation of FGM/C [8, 21]. Previous studies in Sierra Leone have not examined whether women’s empowerment is associated with their intention to circumcise their daughters. In this paper, we explored whether women’s empowerment is associated with their intentions to have their daughters undergo FGM/C. An understanding of women’s intentions to have their daughters undergo FGM/C is important for informing strategies to end the practice.

## Methods

### Data source

Data for this study are from the 2013 Demographic and Health Survey (DHS) of Sierra Leone [7]. The Statistics Sierra Leone (SSL) implemented the survey in collaboration with the Ministry of Health and Sanitation. The 2013 DHS was the second since the inception of the DHS survey in Sierra Leone in 2008. The survey captured information on women’s background characteristics, FGM/C and maternal and child health indicators [7].

To obtain reliable estimates, the sampling took into account rural–urban variations. Sampling was in two stages [7]. In the first step, Primary Sampling Units (PSUs) or clusters were identified from the list of enumeration areas (EAs) generated in the Population and Housing Census. A total of 435 clusters were identified from urban (158) and rural (277) areas. Approximately, thirty households were systematically selected in the second stage in each cluster [7]. All eligible persons in each sampled household were targeted for inclusion in the survey. This comprised women aged 15–49 and men aged 15–59 years. In this study, 7,706 women with complete data on all variables of interest were included in the analysis.

### Measurement of variables

#### Outcome variable

The outcome variable in this study is women’s intentions to circumcise their daughters in future. The intention to circumcise was derived from self-reported response to a question asking women “Do you intend to have daughter(s) circumcised in future?” Possible responses were ‘Yes’, ‘No’, ‘Don’t Know’. All the ‘yes’ responses were coded 1 while ‘no’ responses were coded as ‘0’. All “don’t know” responses were excluded from the sample in order to have a sample with precise data on the intention of women to circumcise their daughters.

#### Individual-level factors

The main explanatory variable for the study was women’s empowerment. Using principal component analysis, this variable was derived from four indicators guided by previous studies [7, 10]. The indicators are: (a) labour force participation (measured by current employment status) [10]; (b) acceptance of wife beating under some circumstances (e.g. neglects children, burns food, refuses to have sex with husband, argues with husband and visitations without permission from husband); (c) knowledge level (e.g. education level, listens, to the radio, reads newspaper and watches television); and (d) decision-making power (decision on respondent’s healthcare, house earning, household purchases and visiting family members). A standardized score with mean 0 and standard deviation 1 was generated, with higher scores being indicative of high disagreement to wife beating, high knowledge, and high decision-making power. We divided the resultant scores into tertiles to allow for nonlinear effects and provided results that were easily interpretable. These were all coded as low, medium and high, representing low, average and high scores respectively. We considered other explanatory variables based on conceptual relevance and previous studies [10, 22, 23]. These are: age (categorised as 15–19, 20–24, 25–29, 30–34, 35–39, 40–44, 45–49 years), household wealth index (poorest, poorer, middle, richer, richest), partner’s education (no education, primary and secondary or higher), and FGM/C status of the respondent (whether the participant was cut or not).

#### Contextual factors

‘Contextual factors’ were considered as circumstances affecting a woman at the household and community level. These are the commonalities and dissimilarities based on an individual’s household or community characteristics and represent clusters in the same family and community. The factors considered at this level are whether FGM/C is required by religion (yes or no), if it is socially accepted (yes or no), family headship (male or female) and place of residence (urban or rural).

#### Modelling approach

We estimated multivariate multilevel logistic regression models to examine the association between the individual and contextual factors associated with women’s intentions to circumcise their daughters. We specified three-level models focusing on women (level 1), contextual factors (level 2) and district variations within the country (level 3). Overall, there were four models, including an empty model devoid of any explanatory variable. This model was specified to decompose the degree of variance between district and contextual level variables. The second and third models comprised individual-level factors only and contextual-level factors only, respectively. The fourth and final model is the full model controlling for both individual and contextual-level factors.

The results are presented as odds ratios (ORs) with their corresponding credible intervals (CrIs). We used Bayesian statistical inference to derive probability distributions of associations with 95% credible instead of confidence intervals [24]. We used median odds ratio (MOR) to measure the plausible contextual effects [25]. We computed the similarity between respondents within the same contextual circumstances and the same district using the Variance Partition Coefficient (VPC). The VPC denotes the proportion of the variance in FGM/C intention related to contextual and district-level factors. Thus, it measures the constellation of likelihood of FGM/C within the same context or district.

The MOR presents the second (contextual) or third (district) district-level factors in terms of odds ratio and accounts for the magnitude of FGM/C intention that is linked to contextual and district level factors. A higher MOR indicates the relevance of contextual factors in influencing women’s FGM/C intentions for their daughters. We investigated multicollinearity between the independent variables using variance inflation factor (VIF) [26]. We found no correlation between the variables, which indicates that the models produce robust results. We used MLwinN software version 3.05 for analysis [27]. We used the Bayesian Deviance Information criterion to measure the extent to which our models fitted the data.

### Ethical approval

Secondary data was used for this study. DHS such as the 2013 Sierra Leone DHS follow protocols for ensuring the protection of privacy of research participants. Further, the Ministry of Health and Sanitation of Sierra Leone and the ethics committee of the DHS Program approved the study. Details about the ethical approval can be accessed from http://goo.gl/ny8T6X.

## Results

### Socio-demographic characteristics of study participants

As illustrated in Table 1, most of the women were participating in labour force (80.5%). About a third of the women had moderate levels of acceptance of wife-beating (39.4%), and moderate decision-making power (35.6%). Distribution by age showed that participants were concentrated around the middle age groups (25–39). About a quarter (23%) of the women were from the poorest households while 67% had partners with no education. Most (79%) of the women were from male-headed households while 63% believed that FGM/C was a religious requirement. More than a third (39%) of the women were from the Northern region while 76% lived in rural areas.

**Table 1:** Socio-demographic characteristics of study participants

Variable	Frequency	Percentage
	(n)	(%)
*Individual level factors*		
Labour force participation		
Yes	5,146	80.5
No	1249	19.5
Wife beating acceptance		
Low	2,066	32.3
Medium	2,519	39.4
High	1,810	28.3
Women’s knowledge level		
Low	2,297	35.9
Medium	2,151	33.6
High	1,946	30.4
Decision making power		
Low	2155	33.7
Medium	2277	35.6
High	1963	30.7
Age		
15–19	187	2.9
20–24	775	12.1
25–29	1,448	22.7
30–34	1,417	22.2
35–39	1,350	21.1
40–44	680	10.6
45–49	537	8.4
Wealth index		
Poorest	1,469	23.0
Poorer	1,428	22.3
Middle	1,406	22.0
Richer	1,194	18.7
Richest	898	14.0
Partner’s education		
No education	4,212	67.0
Primary	562	8.9
Secondary +	1,517	24.1
Circumcised		
No	153	2.4
Yes	6,242	97.6
*Contextual level*		
Social acceptance of FGM/C		
No	2,259	35.3
Yes	4,136	64.7
Required by religion		
No	2,366	37.0
Yes	4,028	63.0
Family head		
Male	5,066	79.2
Female	1,329	20.8
Region		
Eastern	1,672	26.2
Northern	2,513	39.3
Southern	1,488	23.3
Western	722	11.3
Residence		
Urban	1,545	24.2
Rural	4,850	75.8

*Source*: 2013 Sierra Leone Demographic and Health Survey

### FGM/C intention by women’s empowerment and sociodemographic characteristics

A higher proportion of women who participated in labour force intended to circumcise their daughters compared to those who did not participate in labour force (91.2%, CI = 90.4–91.9 and 86.0%, CI = 84.1–87.8, respectively). The proportion of women who intended to circumcise their daughters was significantly higher among those with high acceptance of wife beating than among those with low acceptance of wife-beating (94.9%, CI = 93.8–95.8 and 86.4%, CI = 84.9–87.8, respectively). Similarly, the proportion that intended to circumcise their daughters was significantly higher among those with low than among those with high knowledge levels (91.0%, CI = 89.0–92.8 and 81.6%, CI = 79.8–83.2). There were no statistically significant variations by age in women’s intentions to circumcise their daughters. Variations by household wealth status show that the proportion of women who wanted to perform FGM/C on their children was highest among women in the poorest quintile and lowest among those in the richest quintile (95.3%, CI = 94.1–96.3 vs 71.6%, CI = 68.6–74.4). The proportion of women intending to cut their daughters was, on the other hand, lowest among those whose partners had secondary or higher education (78.6%, CI = 76.5–80.6 and 81.6%, CI = 79.8–83.2, respectively) and highest among those whose partners had no education (94.1% CI = 93.4–94.8). The proportion of women who intended to cut their daughters was highest among those from the poorest households and lowest among those from the richest households (95.3%, CI = 94.1–96.3 and 71.6, CI = 68.6–74.4, respectively) (Table 2).

**Table 2:** Distribution of women intending to cut their daughters by various characteristics

Variable	Proportion Intending to circumcise daughter (95% CI)	*p* value
*Individual level factors*		
Labour force participation (%)		
Yes	91.2 (90.4–91.9)	< 0.001
No	86.0 (84.1–87.8)	
Wife beating acceptance (%)		< 0.001
Low	86.4 (84.9–87.8)	
Medium	89.7 (88.4–90.8)	
High	94.9 (93.8–95.8)	
Women’s knowledge level (%)		< 0.001
Low	93.6 (92.5–94.6)	
Medium	94.5 (93.4–95.4)	
High	81.6 (79.8–83.2)	
Decision making power (%)		< 0.001
Low	91.0 (89.0–92.8)	
Medium	90.0 (87.7–91.9)	
High	85.0 (82.2–87.4)	
Age (%)		0.354
15–19	90.8 (85.8–94.1)	
20–24	91.0 (88.8–92.8)	
25–29	90.6 (89.0–92.0)	
30–34	90.5 (88.9–91.9)	
35–39	89.5 (87.8–91.0)	
40–44	90.1 (87.6–92.2)	
45–49	87.4 (84.3–89.9)	
Wealth index (%)		< 0.001
Poorest	95.3 (94.1–96.3)	
Poorer	93.2 (91.7–94.5)	
Middle	93.5 (92.0–94.7)	
Richer	90.8 (89.1–92.2)	
Richest	71.6 (68.6–74.4)	
Partner’s education (%)		< 0.001
No education	94.1 (93.4–94.8)	
Primary	92.0 (89.5–94.0)	
Secondary +	78.6 (76.5–80.6)	
Circumcised (%)		< 0.001
No	31.6 (24.3–39.9)	
Yes	91.4 (90.6–92.0)	
*Contextual level*		
Social acceptance of FGM/C (%)		< 0.001
No	97.6 (97.1–98.0)	
Yes	76.7 (74.8–78.3)	
Required by religion (%)		< 0.001
No	78.5 (76.7–80.1)	
Yes	96.6 (96.0–97.2)	
Family head (%)		< 0.01
Male	90.7 (90.0–91.5)	
Female	87.9 (86.1–89.5)	
Region (%)		< 0.001
Eastern	91.2 (89.7–92.6)	
Northern	93.8 (92.8–94.7)	
Southern	91.6 (90.1–92.8)	
Western	69.8 (66.2–73.1)	
Residence (%)		< 0.001
Urban	81.4 (79.5–83.0)	
Rural	93.8 (93.1–94.5)	

*Source*: 2013 Sierra Leone Demographic and Health Survey

Contrary to expectations, the proportion of women intending to cut their daughters was highest among those who reported that the practice is not socially acceptable than among those who felt otherwise (97.6%, CI = 97.1–98.0, and 76.7%, CI:74.8–78.3, respectively). The proportion intending to cut their daughters was also higher among women who were of the view that the practice was a religious requirement than among those who had contrary view (96.6%, CI = 96.0–97.2). The proportion was also higher among women in male-headed households than among those in female-headed households (90.7%, CI = 90.0–91.5 and 87.9%,CI = 86.1–89.5, respectively). The proportion intending to circumcise their daughters was highest in the Northern region (93.8%, CI = 92.8–94.7) and among rural women (93.8%, CI = 93.1–94.5).

### District-level clustering in FGM/C intention

All the models showed substantial variation in FGM/C intention across the districts. Variations at the contextual level were relatively lower in all the models compared with those at the district level. The VPC of 17.7 and 32.1 may be more attributable to contextual than district-level factors (Table 3). The results from the MOR, however, indicate that contextual and district-level factors influence FGM/C intention. Results from model 4 show that the average likelihood of a woman intending to cut her daughter increases by 1.5% (1.2–1.9) and 1.6% (1.3–2.0) respectively (Table 3).

**Table 3:** District-level clustering of FGM/C intentions

	Model 1 (Empty Model)	Model 4 (Final Model)
District level		
Variance (95% CrI)	0.9 (0.2–1.5)	0.2 (0.1–0.4)
VPC %, (95% Crl)	17.7 (4.7–26.9)	5.4 (1.2–10.2)
MOR (95% CrI)	2.4 (1.5–3.2)	1.5 (1.2–1.9)
Explained variation (%)	Ref	76.7
Contextual level		
Variance (95% CrI)	0.7 (0.53–0.9)	0.2 (0.1–0.6)
VPC % (95% Crl)	32.1 (17.9–42.1)	11.6 (4.1–23.5)
MOR (95% CrI)	2.2 (2.0–2.4)	1.6 (1.3–2.0)
Explained variation (%)	Ref	67.1

OR, odds ratios; Crls, credible intervals; VPC, variance partition coefficient; MOR, median odds ratio

### Individual and household-level variations in women’s FGM/C intentions

The results in Table 4 show that women who participated in labour force had significantly higher odds of intending to cut their daughters than those who did not (OR = 1.8, Crl = 1.0–3.3). Similarly, women who scored highly in terms of acceptance of wife beating were significantly more likely to intend to cut their daughters than those of contrary opinion (OR = 1.8, Crl = 1.19–2.9). However, women with high knowledge were significantly less likely to intend to cut their daughters than those whose knowledge was low (OR = 0.5, Crl = 0.3–0.6). Compared to women from the poorest households and those whose partners had no formal education, women from the richest households (OR = 0.3, Crl = 0.2–0.6) and those whose partners had secondary or higher levels of education (OR = 0.4,Crl = 0.3–0.6)) were significantly less likely to intend to circumcise their daughters. Circumcised women were more than 20 times likely to intend to circumcise their daughters than uncircumcised women (OR = 23.8, Crl = 11.8–47.8). Women who indicated that FGM/C is socially acceptable (OR = 10.0, Crl = 8.1–12.3) and those who reported that the practice is required by religion (OR = 5.0, Crl = 4.1–6.0) were significantly more likely to intend to cut their daughters than those who felt otherwise. Rural residents were significantly more likely to intend to circumcise their daughters than their urban counterparts (OR = 2.1, Crl = 1.7–2.7).

**Table 4:** Individual and household-level variations in women’s FGM/C intentions

Variable	OR (95% CrI)
**Fixed-effect**	
*Individual level factors*	
Labour force participation	
Yes	1.8* (1.0–3.3)
No	Ref
Wife beating acceptance	
Low	Ref
Medium	1.4* (1.0–1.9)
High	1.8** (1.19–2.9)
Women’s knowledge level	
Low	Ref
Medium	0.6 (0.3–1.3)
High	0.5*** (0.3–0.6)
Decision making power	
Low	Ref
Medium	0.9 (0.6–1.3)
High	0.7* (0.5–0.9)
Age	
15–19	Ref
20–24	0.2* (0.1–0.8)
25–29	0.3 (0.1–1.0)
30–34	0.2* (0.1–0.9)
35–39	0.2* (0.1–0.7)
40–44	0.3* (0.1–0.9)
45–49	0.1** (0.0–0.5)
Wealth index	
Poorest	Ref
Poorer	0.6 (0.3–1.1)
Middle	0.6 (0.3–1.1)
Richer	0.5* (0.2–0.8)
Richest	0.3** (0.2–0.6)
Partner’s education	
No education	Ref
Primary	0.7 (0.4–1.2)
Secondary +	0.4*** (0.3–0.6)
Circumcised	
No	Ref
Yes	23.8*** (11.8–47.8)
*Contextual level*	
Social Acceptance of FGM/C	
No	Ref
Yes	10.0*** (8.1–12.3)
Required by religion	
No	Ref
Yes	5.0*** (4.1–6.0)
Family head	
Male	Ref
Female	0.8 (0.7–1.0)
Residence	
Urban	Ref
Rural	2.1*** (1.7–2.7)
**Random-effect**	
Sample Size	
District level	14
Contextual level	369
Individual level	2,399

OR = Odds ratios, Crls = credible intervals

*p < 0.05, **p < 0.01, ***p < 0.001

### Community-level variations in FGM/C intentions

Labour force participation, acceptance of wife beating, knowledge level, circumcision status, views about social acceptance of FGM/C and residential status were significantly associated with FGM/C intention. In particular, women who participated in labour force (aOR = 2.5, CrI = 1.3–4.7) and those who accepted wife beating (aOR = 2.7, CrI = 1.7–4.5) had significantly higher odds of intending to cut their daughters than those who felt otherwise (Table 5). In contrast, the likelihood of intending to cut their daughters was significantly lower among women with high than among those with low knowledge (aOR = 0.4, CrI = 0.3–0.7). Women aged 45–49 years had significantly lower odds of intending to cut their daughters than those aged 15–19 years (aOR = 0.2, CrI = 0.0–0.6). The odds of FGM/C intention were significantly lower among women whose partners had at least secondary education compared to those whose partners had no formal education (aOR = 0.5, CrI = 0.4–0.8). There was a significantly higher likelihood of FGM/C intention among circumcised than uncircumcised women (aOR = 9.4, CrI = 4.3–20.6). Women who reported that the practice is socially acceptable were ten times more likely to intend to cut their daughters than those who felt otherwise (aOR = 9.9, CrI = 6.7–14.8). The likelihood of intending to cut their daughters was also significantly higher among women who believed that the practice is a religious requirement than those who felt otherwise (aOR = 8.9, CrI = 6.0–13.3).

**Table 5:** Community-level variations in FGM/C intentions

Variable	aOR (95% CrI)
*Individual level factors*	
Labour force participation	
Yes	2.5** (1.3–4.7)
No	Ref
Wife beating acceptance	
Low	Ref
Medium	1.8** (1.2–2.6)
High	2.7*** (1.7–4.5)
Women’s knowledge level	
Low	Ref
Medium	0.7 (0.3–1.6)
High	0.4*** (0.3–0.7)
Decision making power	
Low	Ref
Medium	0.9 (0.5–1.3)
High	0.8 (0.5–1.3)
Age	
15–19	Ref
20–24	0.3 (0.1–1.2)
25–29	0.4 (0.1–1.5)
30–34	0.3 (0.1–1.3)
35–39	0.3 (0.1–1.0)
40–44	0.3 (0.1–1.2)
45–49	0.2** (0.0–0.6)
Wealth index	
Poorest	Ref
Poorer	0.6 (0.3–1.3)
Middle	0.6 (0.3–1.3)
Richer	0.5 (0.3–1.1)
Richest	0.5 (0.2–1.0)
Partner’s education	
No education	Ref
Primary	0.7 (0.4–1.3)
Secondary +	0.5** (0.4–0.8)
Circumcised	
No	Ref
Yes	9.4*** (4.3–20.6)
*Contextual level*	
Social acceptance of FGM/C	
No	Ref
Yes	9.9*** (6.7–14.8)
Required by religion	
No	Ref
Yes	8.9*** (6.0–13.3)
Family head	
Male	Ref
Female	0.79 (0.5–1.2)
*Residence*	
Urban	Ref
Rural	0.7 (0.6–1.8)
**Random-effect**	
District level	
Variance (95% CrI)	0.2 (0.1–0.4)
VPC % (95% Crl)	5.4 (1.2–10.2)
MOR (95% Crl)	1.5 (1.2–1.9)
Explained variation (%)	76.7
Contextual level	
Variance (95% CrI)	0.2 (0.1–0.6)
VPC % (95% Crl)	11.6 (4.1–23.5)
MOR (95% CrI)	1.6 (1.3–2.0)
Explained variation (%)	67.1
Sample size	
District level	14
Contextual level	369
Individual level	2,399

OR, odds ratios; Crls, credible intervals

*p < 0.05, **p < 0.01, ***p < 0.001

## Discussion

The main aim of this study was to investigate the association between women’s empowerment and their intention to circumcise their daughters in Sierra Leone. The findings of this study revealed that women’s empowerment was significantly associated with their intention to circumcise their daughters. Specifically, the intention to circumcise daughters was more prevalent among women who participated in labour force, and those who accepted wife beating than among those who did not.

The findings are consistent with those of previous studies, which have shown that women who participate in labour force, particularly those who are self-employed, and those who accept wife beating are more inclined to cut their daughters than those who do not participate in labour force or those who reject wife beating [28–30]. A previous study showed that women who accept wife beating and other forms of violence have higher chances of experiencing and/or supporting FGM/C than those who reject violence [28]. Being empowered enlightens women and liberates them to exercise their rights, and actively participate in labour force, fortifies their value and equips them to be functional in all spheres of life [36]. Although our finding that women who participated in labour force had significantly higher likelihood of intending to circumcise their daughters than those who did not is inconsistent with previous evidence, it is more plausible in Sierra Leone where FGM/C is culturally entrenched [2, 37]. It could be that since FGM/C is culturally entrenched in Sierra Leone, women who participate in labour force have the resources needed for cutting their daughters. In contrast, the intention of those who do not participate in labour force to not cut their daughters could be due to lack of resources for doing so. The findings suggest a need for information, education and communications campaigns aimed at changing the perceptions of women in labour force and those with negative gender attitudes regarding FGM/C.

The findings further show that women with high knowledge were significantly less likely to indicate that they intend to circumcise their daughters than those whose knowledge was low.

Knowledgeable women are highly empowered to protect themselves and their children from all forms of abuse, violence and barbaric treatment [30, 31]. Similarly, some previous studies have indicated that highly knowledgeable or highly educated women are less likely to have the intention of subjecting their daughters to FGM/C than those whose knowledge is low [8, 32]. As a result, increasing opportunities for girls to acquire higher education has been suggested as a pathway for ending FGM/C and reducing chances that their daughters will undergo the practice, especially in sub-Saharan Africa where FGM/C is prevalent [33–35].

Our findings show that older women were significantly less likely to have the intention to circumcise their daughters compared to younger women. Evidence from Sierra Leone, Ghana and Cote d’Ivoire, however, shows an association between older age and high likelihood of being cut or having an intention to cut daughters [22, 38–40]. Older women in Sierra Leone may be more open to re-evaluating social norms and practices and given their age, and possibly social status, they may have more power to negotiate change than younger women [41]. Some of these older women might have gone through the procedure, and endured its excruciating pains and as such they may not want to perpetuate such bitter experience [42]. This finding implies that interventions to end FGM/C need to be very friendly to younger women. This will substantially persuade them to unlearn their beliefs about FGM/C and relearn that the act is extremely inhuman and has no medical gains to women [2].

There was a significantly higher likelihood of FGM/C intention among circumcised than uncircumcised women. This could be due to differences in cultural connotations regarding the social value of FGM/C between circumcised and uncircumcised women; uncut women may not attach a high social value to the practice [43, 44]. Interventions to end FGM/C in Sierra Leone should prioritise women who have undergone the practice and tailor education and behavioural communication programmes toward these women [34]. Once they appreciate that the act is anti-human and devoid of any medical benefits, they may spare their daughters from undergoing the practice, and support its elimination.

Our findings further show that women who reported that the practice is socially acceptable and those who believed that it is a religious requirement intended to circumcise their daughters compared to those who felt otherwise. Previous studies in Ethiopia and Egypt have similarly shown that FGM/C intention is high when it is socially accepted [45, 46]. The intention to circumcise daughters among such women could be influenced by the social credence associated with FGM/C [47]. The finding suggests a need for state and non-state actors in Sierra Leone to work together with community and religious leaders to address perceptions that perpetuate the practice. Generally, our findings showed that contextual factors were strong influencers of FGM/C intention. This means that intention to circumcise daughters is not only attributable to personal traits of women but also the prevailing norms around the practice.

### Strengths and limitations of the study

A major strength of this paper is that it is based on a nationally representative sample of women aged 15–49 years in Sierra Leone, which makes the findings generalisable among women within that age group in the country. However, the study has some limitations. The study relied on self-reported intentions to cut daughter(s) which may be biased. Due to the generally high prevalence of FGM/C in Sierra Leone, some women may feel compelled to indicate that they intend to circumcise their daughters just to conform to the societal norms, yet this may not be the case. Additionally, the cross-sectional nature of the study does not allow causal inferences to be made regarding the relationship between women’s empowerment, socio-demographic characteristics and women's intention to circumcise their daughters.

## Conclusion

Our study illustrates that intentions of women to circumcise their daughters are inversely associated with high levels of knowledge and rejection of wife-beating. In addition, the intention to circumcise daughters is more likely among circumcised than uncircumcised women, women who participate in labour force than those who do not, and those who believe that the practice is socially acceptable or is a religious requirement compared to those who believe otherwise. The study underscores the need for a review of existing policies and programmes to refocus on women who have undergone the practice, those with low knowledge, those who support wife beating and young women. Such interventions should highlight the adverse implications of the practice by stressing the psychological, health and social implications of FGM/C on its survivors.

## Data Availability

Data for this study were sourced from Demographic and Health surveys (DHS) and available here: http://dhsprogram.com/data/available-datasets.cfm.

## Abbreviations

DHS: Demographic and Health Survey
FGM/C: Female genital mutilation/cutting
SDGs: Sustainable Development Goals
WHO: World Health Organization
